# Influencing Factors of Dyadic Coping Among Infertile Women: A Path Analysis

**DOI:** 10.3389/fpsyt.2022.830039

**Published:** 2022-03-28

**Authors:** Nan Tang, Yingying Jia, Qing Zhao, Huihui Liu, Junzheng Li, Hongchen Zhang, Lin Han, Chaoji Huangfu

**Affiliations:** ^1^Center for Evidence-Based Nursing, School of Nursing, Lanzhou University, Lanzhou, China; ^2^Center for Disease Control and Prevention, Western Theater Command, Lanzhou, China

**Keywords:** dyadic coping, infertile women, path analysis, family cohesion and adaptability, multiple linear regression analysis

## Abstract

**Background:**

The infertility prevalence of married couples in China is increasing gradually. The dyadic coping level and its influencing factors of infertile women in China are poorly reported. The relationship between dyadic coping and the family cohesion and adaptability in infertile women was investigated.

**Methods:**

A total of 482 infertile women in the reproductive clinics of three affiliated hospitals of the Lanzhou University were selected by the convenience sampling method. The self-made general information questionnaire, family adaptability and cohesion evaluation scale, and dyadic coping questionnaire were used in this study.

**Results:**

The average age of infertile women was 31.73 ± 4.57 years, the duration of infertility was 28.66 ± 27.99 months, the total score of dyadic coping was 132.66 ± 25.49, the total score of family cohesion and adaptability was 101.48 ± 20.96. A significant positive correlation between dyadic coping and family cohesion and adaptability was observed (*r* = 0.74, *p* < 0.01). The multiple linear regression analysis showed that religious belief, number of miscarriages, relationship between family members, family intimacy, and adaptability were the influencing factors of dyadic coping level in the family of infertile women (*R*^2^ = 0.566, *p* < 0.01).

**Conclusions:**

The dyadic coping level of infertile women is in the medium level, which is significantly positively correlated with family intimacy and adaptability. In clinical nursing, nurses try to improve the family relationship of patients to increase the level of dyadic coping of infertile women.

## Background

Infertility is defined by the inability to conceive after 1 year or more of regular unprotected sexual intercourse (1, 2). Currently, the definition of infertility has been enlarged to a wider scope that influences the ability of reproduction of partners (3). It is believed that 12.4% of the couples in the world had trouble achieving pregnancy in 2010 (4). The infertility prevalence of married couples in China accounts for 15%, and it is increasing gradually (5, 6). Infertility is believed to be one of the most stressful life events (7).

Assisted reproductive technology (ART) treatment has been widely applied to help infertile couples achieve a pregnancy. ART contains several types of techniques including ovulation induction (OI), artificial insemination (AI), *in vitro* fertilization (IVF), gamete intrafallopian transfer (GIFT), and intracytoplasmic sperm injection (ICSI). It was reported that the proportion of ART babies accounted for 2.4% of the total babies in 2017 (8).

Infertility could lead to emotional, social, sexual, and family relationship problems, which not only exert a negative impact on the relationship of the partners, but also reduce the therapeutic effect of the ART treatment (9, 10). Some negative feelings such as isolation, anxiety, guilt, and depression are common in the infertile couples. The previous study indicated that infertility brought more stress for women than men for the reason that females have to experience maximum treatment steps (11).

Dyadic coping means partners support each other through individual and joint efforts during the disease stress (12, 13). It was reported that family functions and the level of dyadic coping between a husband and wife will affect their mental health and life quality (14, 15). Dyadic coping includes stress communication, supportive coping, delegated coping, joint coping, negative coping, and coping quality evaluation. The individual efforts and the cooperation of the husband and wife are considered. It was reported that men prefer problem-focused coping strategies, but women prefer emotion-focused coping methods (4). When the individuals provide sufficient dyadic coping support to each other, it can improve the trust, security, and intimacy of both sides and have a positive impact on the relationship between a husband and wife, which is beneficial to the physical and mental health of both (16). Therefore, couples can deal with the disease as a whole and not just as individuals. We will explore the factors affecting the dyadic coping of infertile patients, which will help to formulate targeted intervention strategies.

The dyadic coping between infertile couples is a complex process, which may be affected by many factors. First, the sociodemographic characteristics of the patients (age, educational background, family environment, occupation, income, religious belief, etc.) will affect the coping ability of the couples. The older infertile couples will face a heavier reproductive burden, and they are more inclined to negative coping styles. Couples with higher education and better economic status have a higher cognition of the disease and can better participate in treatment decision-making. Religious belief is related to the mental health and quality of life among infertile women. Religious belief can provide coping strategies such as optimism, supportive relationship, gratitude and appreciation for marital life, and spiritual resonance and affect their treatment attitude (17).

Meanwhile, from the perspective of a disease, the years and causes of illness, whether they have received treatment, and the outcome of the treatment are closely related to the coping style of the patients. The study found that the coping style of infertile women will change with the development of the disease. At the initial stage of the diagnosis, most infertile women showed a positive attitude and a high compliance rate. In the middle of the course, they turned toward a negative-coping psychology. As the course of the disease extended, their coping styles changed again (18, 19). In addition, the more abortions the patients experience, the more difficult it is for them to get pregnant. Abortion is a common experience of infertile women and their spouses. The joint coping ability of the husband and wife will directly affect the marital relationship (20). These factors are worth exploring and their relationship with dual coping.

A series of studies show that dyadic coping is closely related to psychological distress, marital relationship, and quality of life (21, 22). The dyadic coping of couples is to work together to cope with the pressure after sensing each other's pressure. The goal of dyadic coping is to maintain or reconstruct family function, release their own burden, rebuild the partnership, make both sides trust and help each other, increase the sense of security, and actively rebuild an intimate relationship. Dyadic coping can reduce the level of psychological distress of the couples (23), which is a protective factor of marital relationship and intimacy. The previous studies reported the application of dyadic coping in the infertile population. For example, the role of dyadic coping on the marital adjustment of partners undergoing ART treatment was investigated (4). Thus, the marital relationship will directly affect the coping style and ability of both the husband and wife.

It has been established that infertility has a negative impact on marital relationships and sexual relationships in Poland (20). Meanwhile, studies show that infertility in women of Iran (24), Ghana (25), and India (26) report lower relationship satisfaction than fertile women. A study from Milan proved that scores on positive dyadic coping styles contributed to higher marital adjustment, and the promoting effect of husband and wife's reciprocal supportive behaviors on positive dyadic coping (4). An Israeli study shows that a dyadic approach to studying illness perceptions can uncover patterns of couples at risk for poor adjustment (27). The previous studies mainly focused on the impact of dyadic coping on marital relationship and spousal support. In this study, we considered both the couples and other family members factors. According to the previous studies, the degree of support and coping provided by the spouses and perceived by the patients was different. We believe that it is important to study the influencing factors of dyadic coping perceived by the patients.

Family is an important environment that affects the development of an individual's body and mind. Family cohesion and adaptability are two important indicators of family function. They can reflect the close relationship of family members and the ability of the family to deal with major events (28, 29). The improvement of family adaptability and cohesion could promote treatment effect and reduce the stress of the patients (30). Family adaptability is viewed as the ability of responding to situation changes and sudden stress. Family cohesion is defined as the emotional relationship among family members (31). Research shows that the effect of psychological counseling for both the husband and wife is better than the unilateral intervention of the patients. It is confirmed that the synchronous intervention between husband and wife will be more helpful to promote sharing and mutual support between husband and wife, and can improve the coping ability and psychological conditions of both sides. However, a few studies apply family adaptability and the cohesion evaluation scale to Chinese infertility populations, and analyze the potential influencing factors.

To sum up, the dyadic coping ability of infertile women is affected by physiological, psychological, social, and other factors. The role of various factors should be considered to improve the dyadic coping level. The factors that may affect the dyadic coping of infertile women are sociodemographic factors, disease-related factors, and family relations.

The dyadic coping of an infertility patient is a complex process. Religious belief is related to the mental health and quality of life among infertile women. Religious belief can provide coping strategies such as optimism, supportive relationship, gratitude, and appreciation for marital life and spiritual resonance, and affect their treatment attitude (17). Meanwhile, from the perspective of the disease, the more miscarriages patients experience, the more difficult it is for them to get pregnant. A miscarriage is a common experience of infertile women and their spouses. The common coping ability of the husband and wife will directly affect the marital relationship (20). Therefore, we want to further explore the influence of religious beliefs and number of miscarriages on the dyadic coping of the infertility patient.

In this study, we stated the following hypotheses:

Hypothesis 1. Family cohesion and adaptability, relationship with family members, religious belief, and number of miscarriages have a significant impact on the dyadic coping of the infertility patient.

Hypothesis 2. Family cohesion plays a mediating role between relationship with family members and dyadic coping.

Hypothesis 3. Family adaptability plays a mediating role between relationship with family members and dyadic coping.

In this study, we investigated the dyadic coping level and its influencing factors in infertile women according to these three aspects. The relationship between the dyadic coping level and family cohesion and adaptability were explored. This study might provide evidence for the development of husband and wife-centered intervention program of infertile women.

## Materials and Methods

### Participants

From July to October 2020, the infertile women taking ART treatment in the reproductive clinics of three affiliated hospitals of the Lanzhou University were selected using the convenience sampling method. We had access to information that could identify the individual participants during data collection. Inclusion criteria: female patients diagnosed as infertile; patients voluntarily participating in this study; patients having the ability to read and communicate normally. Exclusion criteria: patients with a history of mental illness, cognitive impairment, or other diseases, and unable to fill in the questionnaires. The patients with cancer or other systemic diseases were excluded. A total of 482 infertile women were enrolled in this research. All the participants signed the informed consent and agreed to participate in this research. The mean age of the infertile women was 31.73 ± 4.57 years, and the average duration of infertility was 28.66 ± 27.99 months; 22.4% of the patients lived in the rural areas, 36.9% lived in towns, and 40.7% lived in the urban areas.

### Measures

#### General Information Questionnaire

A self-made general information questionnaire was used in this study. The questionnaire mainly includes the demographic characteristics and disease-related information of the patients. The demographic characteristics contain age, nationality, education level, occupation, per capita monthly income of the family, etc. The disease-related information includes the causes of infertility, duration of infertility, whether or not they received assisted-reproductive therapy previously, and the main causes of reproductive pressure.

#### Family Adaptability and Cohesion Evaluation Scale, Second Edition

Family adaptability and cohesion evaluation scale, second edition (FACES-II), designed by Olson, contains family adaptability and family cohesion and includes 30 items. The test–retest reliability of the two parts was 0.84 and 0.54, and the internal consistency was 0.85 and 0.76, respectively. Of the two major dimensions, the essence of cohesion is sought through questions such as “family members know each other's close friends” and “our family does things together,” whereas adaptability is explored through questions such as “when problems arise we compromise” and “family members say what they want.” The questions offer both positive and negative aspects of family life, for instance “it is easier to discuss problems with people outside the family than with other family members,” can be contrasted with “family members discuss problems and feel good about the solutions.” A five-point Likert scale ranging from “1” to “5” was used in FACES-II. The points from “1” to “5” represent “never,” “occasionally,” “sometimes,” “often,” and “always.” The higher the score, the better the family cohesion and adaptability. Based on the score, the family adaptability degree was divided into irregular, flexible, regular, and rigid. The cohesion degree was divided into entanglement, intimacy, freedom, and looseness. The 4 kinds of adaptability levels and 4 types of intimacy levels were combined to form 16 types in total. When a couple is at the extreme level, the highest or lowest level, the family is an extreme family and the family function is seriously abnormal. If the couple is in the middle level, it indicates that this is a balanced family and the family function is normal. The rest are intermediate families, which represent an abnormal family function. The scale shows good internal consistency (Cronbach's alpha = 0.943).

#### Dyadic Coping Inventory

The dyadic coping inventory (DCI), designed by Bodenmann, detects dyadic coping behaviors using 37 items (32, 33). It can be used to evaluate the stress communication between the subjects and their spouses, the supportive coping provided by the subjects themselves, and the quality of supportive coping from their spouses. In this study, the Chinese version of DCI in 2016, which has been cross-culturally adjusted, has a good construct validity, and the Cronbach's α is 0.51–0.80. The DCI includes 6 dimensions: stress communication, supportive coping, delegated coping, joint coping, negative coping, and coping quality evaluation. Stress communication refers to expressing the pressure (e.g., “I show my partner through my behavior when I am not doing well or when I have problems.”); supportive coping refers to helping, understanding, and comforting each other (“I express to my partner that I am on his/her side”); delegated coping refers to taking the responsibility of the other (“When my partner feels he/she has too much to do, I help him/her out.”); negative coping refers to providing hostile, contradictory, or superficial help, or not caring (“I do not take my partner's stress seriously.”), Common coping refers to the use of joint cooperation, such as solving problems together (“We engage in a serious discussion about the problem and think through what has to be done.”). A Likert type five-point scale ranging from “1” (never) to “5” (very often) was applied in the DCI. The higher the score, the more supportive was the coping of the couple. The score of negative coping items needs to be reversed. This scale indicates the dyadic coping methods of couples. The higher the score, the more the couples feel they are dealing with the stressful conditions together. The cutoff scores are established in the DCI as follows: dyadic coping below average (DCI total score: <111), dyadic coping in the normal range (DCI total score: 111–145), and dyadic coping above average (DCI total score: >145). The questionnaire presents a good internal consistency (Cronbach's alpha = 0.881).

#### Procedure

The study was approved by the Ethical Committee of Nursing School, Lanzhou University. The study was conducted in accordance with the Declaration of Helsinki. A number of five trained undergraduate nursing students served as investigators. They first explain the purpose of the survey to the respondents. All participants signed the informed consent forms, then the respondents independently filled in the questionnaire according to their actual situation under the guidance of the investigators. A total of 500 questionnaires were distributed, of which 482 were valid, and the effective rate was 96.4%. The valid questionnaires were collected after on-site verification. Data confidentiality and anonymity were ensured.

### Data Analysis

Epidata 3.0 software was used to establish the database and data entry. SPSS23.0 software was used to perform the *t*-test, variance analysis, Pearson's correlation analysis, and multiple linear regression analysis. Amos 23.0 software was used to build the fitting model of path analysis, and the maximum likelihood ratio method was used to modify and fit the model. The alpha level was 0.05.

## Results

### Demographic Characteristics and Dyadic Coping Score of Infertile Women

A total of 482 infertile women aged 31.73 ± 4.57 were investigated in this study, and 96.1% of them were from Han (Table 1). About 64.3% of them had a bachelor's degree or above, and most of them were urban residents, and rural residents accounted for only 22.4%. Only 9.96% of the infertile women had a monthly income of more than 10,000 yuan. More than half of the investigated patients had been suffering from infertility for 2–5 years, and 42.3% of the patients had been diagnosed as infertile for <1 year. Around 42.3% of the patients had been treated previously.

**Table 1:** Univariate analysis of dyadic coping score of infertile women (*n* = 482).

**Variables**		**Number**	**Score (Mean ±SD)**	***F*/*t***	**Effect size**	***P***
Age (years)	≤ 30	234	140.45 ± 22.94	31.02	0.163^a^	*P* < 0.01
	31~35	148	131.27 ± 22.31			
	36~40	83	111.89 ± 26.62			
	>40	17	138.82 ± 21.95			
Nationality	Han	463	132.54 ± 25.28	0.25	0.002^a^	0.862
	Hui	16	133.75 ± 33.27			
	Tibetan	2	146.00 ± 9.90			
	Tu	1	143.00 ± 0.00			
Education level	Primary school	16	136.56 ± 18.85	25.03	0.173^a^	*P* < 0.01
	Junior school	77	112.23 ± 26.56			
	Senior School	79	123.84 ± 23.98			
	College	310	139.74 ± 22.30			
Residence place	Rural areas	108	119.55 ± 25.46	28.86	0.108^a^	*P* < 0.01
	Towns	178	131.10 ± 24.47			
	Urban areas	196	141.29 ± 23.05			
Occupation	Farmer	64	118.53 ± 26.87	8.75	0.068^a^	*P* < 0.01
	Teacher or staff	72	141.36 ± 19.84			
	Public servant or manager	69	136.04 ± 22.04			
	Medical worker	45	139.40 ± 21.12			
	Other	232	131.53 ± 26.74			
Per capita monthly income of the family (yuan)	≤ 3,000	121	120.27 ± 26.28	24.69	0.134^a^	*P* < 0.01
	3,000~5,000	202	131.18 ± 22.66			
	5,001~10,000	111	141.26 ± 22.85			
	≥10,000	48	150.17 ± 23.97			
Religious belief	No	472	132.58 ± 25.59	6.391	0.907^b^	0.012
	Yes	10	150.30 ± 10.40			
Years of marriage (years)	≤ 3	189	142.47 ± 22.29	31.77	0.166^a^	*P* < 0.01
	4~6	142	134.80 ± 21.66			
	7~10	90	121.56 ± 24.79			
	>10	61	113.66 ± 27.79			
Whether the patient is the only child	Yes	81	139.35 ± 24.29	0.044	0.323^b^	0.833
	No	401	131.30 ± 25.54			
Whether the husband is the only child	Yes	119	137.78 ± 25.30	0.009	0.268^b^	0.926
	No	363	130.98 ± 25.36			
Relationship with family members	Very bad	55	111.20 ± 27.22	57.38	0.325^a^	*P* < 0.01
	Bad	25	104.04 ± 25.61			
	Normal	63	116.89 ± 21.77			
	Good	173	134.56 ± 16.10			
	Very good	166	148.07 ± 22.19			
Duration of infertility (years)	≤ 1	204	135.87 ± 22.08	7.313	0.044^a^	*P* < 0.01
	2~5	244	131.01 ± 27.06			
	6~10	30	131.17 ± 27.03			
	>10	4	80.50 ± 15.00			
Have you ever been treated	Yes	315	132.27 ± 26.51	1.60	0.044^b^	0.207
	No	167	133.38 ± 23.50			
Have you ever took *in vitro* fertilization-embryo transfer treatment	Yes	212	133.95 ± 24.35	0.452	0.091^b^	0.502
	No	270	131.64 ± 26.35			
Pregnancy experience	Yes	174	131.25 ± 25.29	0.05	0.091^b^	0.832
	No	308	133.57 ± 25.62			
Times of abortion	0	308	133.67 ± 25.88	3.905	0.016^a^	0.021
	1~2	163	132.13 ± 23.10			
	3~4	11	112.09 ± 39.21			
Sources of fertility stress	Parents-in-law	83	125.12 ± 28.09	6.23	0.050^a^	*P* < 0.01
	Parents	33	144.70 ± 23.53			
	Spouse	38	135.29 ± 20.10			
	Oneself	257	130.85 ± 26.37			
	Classmates, friends, colleagues, neighbors	71	140.99 ± 17.59			
Cause of illness	Oviduct	163	139.33 ± 22.05	6.471	0.064^a^	*P* < 0.01
	Ovary	51	126.55 ± 31.13			
	Uterus/cervix	14	110.71 ± 30.84			
	Genetic/immune diseases	10	143.40 ± 19.25			
	Other	102	132.66 ± 25.49			

a *Used partial η^2^ as effect size*.

b *Used Cohen's d as effect size*.

The total score of dyadic coping was 132.66 ± 25.49, which was in the middle level. Communication and perceptions about the quality and quantity of partner support are listed in Table 2. The score of stress communication of infertile women was 14.09 ± 3.69 and that of the patients' perceived spouse coping was 14.11 ± 3.70. There was no significant difference in stress communication between the patients and the patients' perceived spouse coping (*t* = 0.589, *p* = 0.556). The score of supportive coping of infertile women was 18.04 ± 4.39 and that of the patients' perceived spouse coping was 16.98 ± 4.86. The *t*-test showed that there was a significant difference in supportive coping between patients and the patients' perceived spouse coping (*t* = 7.318, *p* < 0.01). The scores of delegated coping of the patients and patient's perceived spouse were 7.29 ± 1.76 and 7.27 ± 1.81, respectively. In addition, the scores of negative coping of infertile women and patient's perceived spouse were 14.99 ± 3.95 and 14.76 ± 3.92, respectively. No significant difference was observed in terms of delegated coping and negative coping between the patients and the patients' perceived spouse coping.

**Table 2:** The scores of different dimensions of infertile women perception (*n* = 482).

**Item**	**Score (Mean ±SD)**	**Item**	**Score (Mean ±SD)**
Stress communicated by oneself	14.11 ± 3.70	Delegated dyadic coping of the partner	7.27 ± 1.81
Stress communication of the partner	14.11 ± 3.70	Negative dyadic coping by oneself	14.99 ± 3.95
Supportive dyadic coping by oneself	18.04 ± 4.39	Negative dyadic coping by partner	14.76 ± 3.92
Supportive dyadic coping of the partner	16.98 ± 4.86	Common dyadic coping	17.98 ± 4.66
Delegated dyadic coping by oneself	7.29 ± 1.76	Evaluation of dyadic coping	7.19 ± 1.96

### Univariate Analysis of Dyadic Coping Score in Infertile Women

A univariate analysis of dyadic coping score in infertile women was performed in this study (Table 1). We found that the scores of age, education level, residence, occupation, monthly income per capita of the family, religious belief, marriage years, relationship with family members, duration of infertility, number of abortions, sources of birth pressure, and causes of disease were statistically different (*p* < 0.05).

### The Relationship Between Dyadic Coping and Family Adaptability and Cohesion Evaluation of Infertile Women

The total score of family cohesion and adaptability of infertile women was 101.48 ± 20.96, of which the score of family cohesion was 54.24 ± 11.23 and the score of adaptability was 47.24 ± 10.30. The family cohesion and adaptability of infertile women were positively correlated with the level of dyadic coping (*r* = 0.741, *p* < 0.01). A significant positive correlation between stress communication of partners and patients and family cohesion (*r* = 0.568, *p* < 0.01) and adaptability (*r* = 0.619, *p* < 0.01) was observed (Table 3). Meanwhile, supportive dyadic coping by oneself, supportive dyadic coping of the partner, delegated dyadic coping by oneself, delegated dyadic coping of the partner, and negative dyadic coping by the partner were also positively correlated with family cohesion and adaptability. In addition, remarkable positive correlation between negative dyadic coping by oneself (*r* = 0.090, *p* < 0.05) and family cohesion was observed (Table 3).

**Table 3:** Correlation between dyadic coping and family adaptability and cohesion.

**Dismensions**	**Stress communication of the partner**	**Stress communicated by oneself**	**Supportive dyadic coping by oneself**	**Supportive dyadic coping of the partner**	**Delegated dyadic coping by oneself**	**Delegated dyadic coping of the partner**	**Negative dyadic coping by oneself**	**Negative dyadic coping by partner**
Adaptability	0.619^**^	0.619^**^	0.666^**^	0.679^**^	0.677^**^	0.631^**^	−0.009	0.105^*^
Cohesion	0.568^**^	0.568^**^	0.671^**^	0.663^**^	0.676^**^	0.633^**^	0.090^*^	0.156^**^

* *p < 0.05*.

** *p < 0.01*.

*Positive copings include spouse stress communication, self stress communication, self supportive coping, spouse supportive coping, self delegated coping and spouse delegated coping; Negative copings include self negative coping and spouse negative coping*.

**Figure 1:**
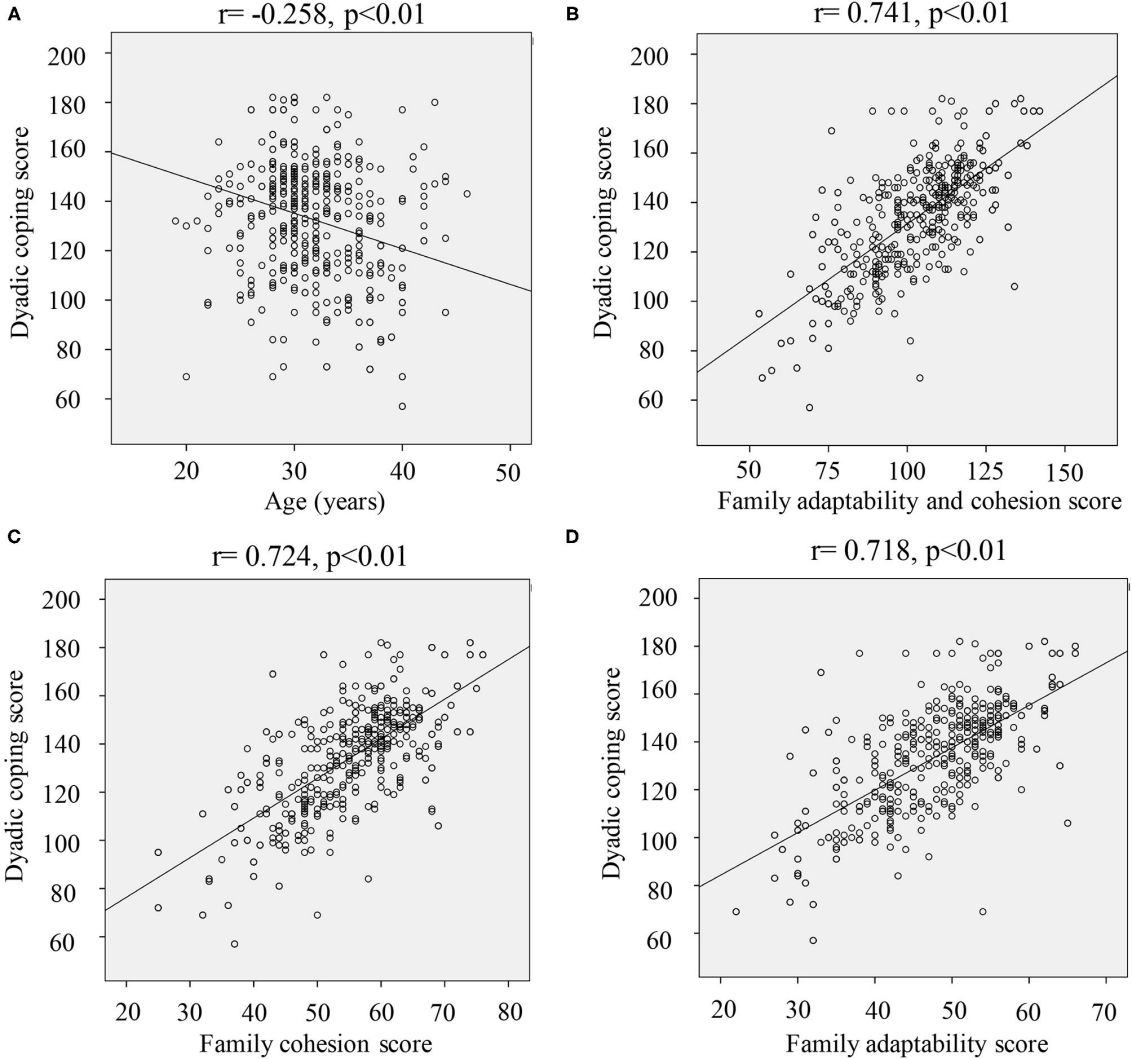
Correlation analysis of the dyadic coping score and other factors. **(A)** Correlation analysis of dyadic coping and age; **(B)** correlation analysis of dyadic coping and family adaptability and cohesion; **(C)** correlation analysis of dyadic coping and family cohesion; **(D)** correlation analysis of dyadic coping and family adaptability.

Meanwhile, there was a significant negative correlation between dyadic coping and age in infertile women (*r* = −0.258, *p* < 0.01) (Figure 1A). However, significant positive correlation between dyadic coping and family cohesion and adaptability score (*r* = 0.741, *p* < 0.01) was observed (Figure 1B). In addition, remarkable positive correlation between family cohesion (*r* = 0.724, *p* < 0.01) (Figure 1C), or family adaptability (*r* = 0.718, *p* < 0.01) (Figure 1D) was also found.

### Influencing Factors of Dyadic Coping Style in Infertile Women

To further explore the influencing factors of dyadic coping style in infertile women, multiple linear regression analysis was performed. The dyadic coping score was set as a dependent variable; the variables with statistical significance in univariate analysis and family cohesion and adaptability were set as independent variables. We found that religious belief, relationship with family members, abortion times, cohesion, and adaptability entered the regression equation (Table 4). The results of the *F*-test showed that *F* = 53.355, *p* < 0.01, indicating that the fitting equation was statistically significant. *R*^2^ = 0.760 and adjusted *R*^2^ = 0.566 indicated that the four independent variables explained 76.0% of the variance variability. The influence factors of infertile women dyadic coping from strong to weak were cohesion and adaptability, abortion times, relationship with family members, and religious belief (Table 4).

**Table 4:** Multiple linear regression analysis of patients' dyadic coping score (n = 482).

**Item**	***B***	***SE***	**β*^′^***	***t***	***p***
Constant	35.363	8.881	-	3.982	<0.01
Religious belief	10.745	5.431	0.060	1.978	0.048
Relationship with family members	1.743	0.867	0.088	2.010	0.045
Times of abortion	−5.750	1.509	−0.120	−3.810	<0.01
Adaptability and cohesion	0.835	0.053	0.686	15.895	<0.01

*R^2^ = 0.760, after adjustment R^2^ = 0.566, F = 53.355, P < 0.01*.

### Path Analysis of Influencing Factors of Dyadic Coping in Infertile Women

**Figure 2:**
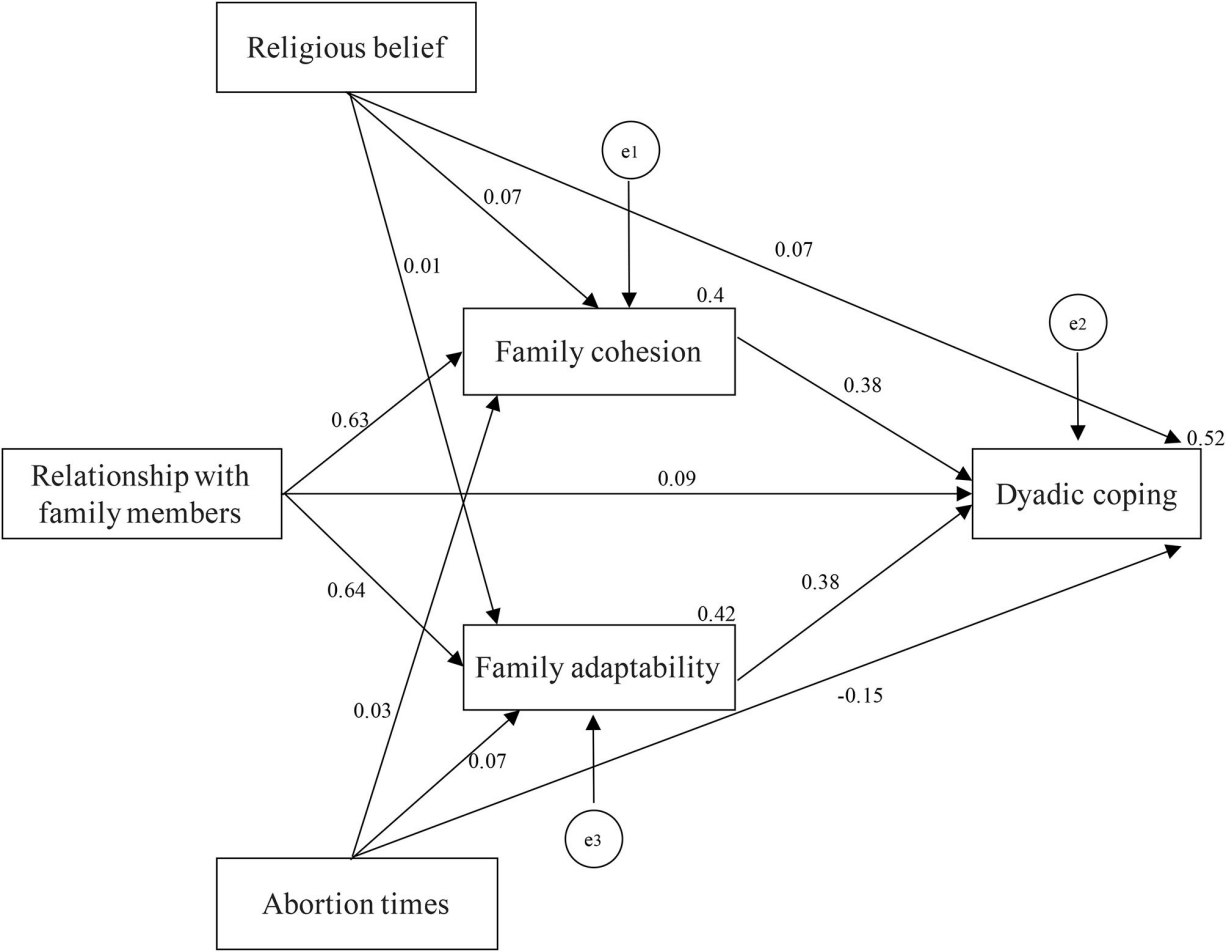
Final model for family adaptability and cohesion, relationship with family members, religious beliefs, number of abortions, and dyadic coping. e1, e2, and e3 represent residual errors for the family cohesion, dyadic coping, and family adaptability, respectively.

The hypothesized path model was made based on the influencing factors entering the regression equation (Figure 2). Standardized regression coefficient was viewed as the path coefficient. Religious belief, abortion times, relationship with family members, family cohesion, and family adaptability directly influenced dyadic coping, and the path coefficients were 0.07, −0.15, 0.09, 0.38, and 0.38, respectively. Meanwhile, religious belief, abortion times, and relationship with family members influenced dyadic coping indirectly through family cohesion, and the path coefficients were 0.07, 0.63, and 0.03, respectively. In addition, religious belief, abortion times, and relationship with family members influenced dyadic coping indirectly through family adaptability, and the path coefficients were 0.01, 0.64, and 0.07, respectively (Figure 2). The analysis data suggested that χ^2^/*df* = 0.678, GFI = 1.000, AGFI = 0.997, NFI = 0.998, CFI = 1.000, RMSEA = 0.000 (Table 5). Each fitting index was in the acceptable range, which indicated that the model had a good fitting degree and was reasonable.

**Table 5:** Model fitting index.

**Statistical test**	**χ^2^/*d83***	**RMSEA**	**GFI**	**AGFI**	**NFI**	**CFI**	**RFI**
Fit index	0.678	0.000	1.000	0.997	0.998	1.000	0.992
Fit standard	<3.00	<0.08	>0.90	>0.90	>0.90	>0.90	>0.90

## Discussion

In this study, the total score of dyadic coping was 132.66 ± 25.49, which was in the middle level. The reason for the low score may be that the treatment and rehabilitation of infertility bring great pressure to infertile couples, which reduces the quality of their reproductive life and the level of dyadic coping (34). In addition, this study showed that the scores of both the supportive and delegated coping of infertile women were higher than their perceived scores of partners' supportive and delegated coping, which is in line with previous study (35). Compared with men, women have stronger communication skills. At home, women may care more about families and help family members as much as possible. This study indicates that the scores of negative coping of infertile women was higher than their perceived partners' negative coping. This might be because a Chinese family is usually influenced by the traditional concept that childbearing is the responsibility of women. Therefore, women will bear great mental pressure, and show more negative response. Positive dyadic coping reduces the financial burden and improves the relationship between couples (28, 36). Therefore, clinical nurses need to improve the ability to identify positive coping, give specific guidance, strengthen positive coping, reduce negative coping, increase mutual support between husband and wife, and improve marital relationship to improve the level of dyadic coping of infertile women.

We found that the score of dyadic coping of the patients with religious beliefs was higher than that of the patients without religious beliefs. The previous study indicated that religious beliefs could provide spiritual resonance for the patients, so they could respond with optimism and gratitude during both marriage and disease treatment. Therefore, the infertile patients with religious beliefs can better adjust their relationship in marriage, and the couples are more likely to take a positive supportive coping facing disease and treatment.

There was a positive correlation between relationship of family member and dyadic coping level of infertile women. The better the relationship between family members, the better the family intimacy and adaptability, which leads to a better family function. Meanwhile, couples have a better ability to support each other and solve problems. Women who believe in better family relationship are more likely to look forward to becoming mothers. Then, they show more cooperation and persistence during treatment, and they also have more positive coping strategies. The support of family members, including psychological and financial support, could enhance the confidence of the patients to overcome the disease (37). They tend to take more positive coping methods, and higher dyadic coping level are achieved. Therefore, the medical staff should pay attention to the family relationship of the patients, fully mobilize the support of the patients' partners and other family members, encourage the patients to communicate with their families and seek help, which might help to alleviate their psychological burden, and adopt positive dyadic coping strategies.

The number of abortions in infertile women was negatively correlated with the dyadic coping style. The more the number of abortions, the lower the level of dyadic coping. This may be because diseases lead to a decline in their self-esteem and fear of social and family rejection, so they tend to adopt more passive coping methods. Therefore, the medical staff should fully understand the patient's treatment history and provide a more specific psychological support and nursing intervention.

A strong positive correlation between the dyadic coping level and family cohesion and adaptability of infertile women was observed. Meanwhile, family cohesion and adaptability were positively correlated with stress communication of the partner, stress communicated by oneself, supportive dyadic coping by oneself, supportive dyadic coping of the partner, delegated dyadic coping by oneself, and delegated dyadic coping of the partner. Stress communication is a way to release negative emotions, which is closely related with cohesion. It is beneficial to release stress, produce more positive emotion, and promote the family cohesion and adaptability if the patients and their spouses have a strong ability to communicate. A good level of dyadic coping plays an important role in maintaining the relationship between the couples. The higher score of positive coping indicates that the couples are more willing to help each other, they have a better quality of life, and higher family cohesion (36). Therefore, nurses should view family cohesion and adaptability as a breakthrough point to strengthen the psychological nursing of infertile couples. In addition, the couple should be encouraged to communicate with each other to improve the positive level of dyadic coping.

Some limitations affected the research. First, the samples of this study came from the tertiary hospitals in the same area, and the sample size was small. Second, with the treatment and rehabilitation of infertility, the relationship between dyadic coping and family cohesion and adaptability will change dynamically. This study has not yet described the dynamic changes of the relationship between the two concepts.

## Conclusion

This study proved that the dyadic coping level of infertile women was moderate, and its influencing factors include religious beliefs, the relationship between patients and family members, abortion times, family cohesion, and adaptability. In clinical work, the nursing staff should take the husband and wife as the center to carry out corresponding intervention and guidance, and strengthen health education and psychological nursing for infertile couples. Second, the nurses should improve the ability to identify positive coping and guide infertile couples to strengthen positive coping. Group counseling such as listening, communication, and sharing could be used to promote the interaction and mutual assistance of the group members. Using these methods will help to improve the dyadic coping of infertile women and effectively solve their psychological problems.

## Data Availability Statement

The original contributions presented in the study are included in the article/supplementary material, further inquiries can be directed to the corresponding authors.

## Ethics Statement

The studies involving human participants were reviewed and approved by the Ethical Committee of Nursing School, Lanzhou University. The patients/participants provided their written informed consent to participate in this study.

## Author Contributions

NT, HZ, LH, and CH designed the study and wrote and revised the manuscript. YJ, QZ, HL, HZ, LH, and JL distributed questionnaires and analyzed data. All authors contributed to the article and approved the submitted version.

## Funding

This study was funded by the Research Project of School of Nursing, Lanzhou University (LZUSON202009), the Natural Science Foundation of Gansu Province (21JR7RA508), and the Fundamental Research Funds for the Central Universities (lzujbky-2021-34).

## Conflict of Interest

The authors declare that the research was conducted in the absence of any commercial or financial relationships that could be construed as a potential conflict of interest.

## Publisher's Note

All claims expressed in this article are solely those of the authors and do not necessarily represent those of their affiliated organizations, or those of the publisher, the editors and the reviewers. Any product that may be evaluated in this article, or claim that may be made by its manufacturer, is not guaranteed or endorsed by the publisher.

## Abbreviations

ART: Assisted reproductive technology
OI: ovulation induction
AI: artificial insemination
IVF: *in vitro* fertilization
GIFT: gamete intrafallopian transfer
ICSI: intracytoplasmic sperm injection
FACES-II, family adaptability and cohesion evaluation scale: second edition
DCI: dyadic coping inventory.
